# Editorial: Targeting Angiogenesis to Treat Autoimmune Diseases and Cancer

**DOI:** 10.3389/fimmu.2020.01005

**Published:** 2020-05-21

**Authors:** Michal A. Rahat, Julia Kzhyshkowska, Vijaya Iragavarapu-Charyulu

**Affiliations:** ^1^Immunotherapy Laboratory, Carmel Medical Center, Haifa, Israel; ^2^The Ruth and Bruce Rappaport Faculty of Medicine, Technion-Israel Institute of Technology, Haifa, Israel; ^3^Institute of Transfusion Medicine and Immunology, Medical Faculty Mannheim, University of Heidelberg, Mannheim, Germany; ^4^German Red Cross Blood Service Baden-Württemberg—Hessen, Mannheim, Germany; ^5^Laboratory of Translational Cellular and Molecular Biomedicine, National Research Tomsk State University, Tomsk, Russia; ^6^Department of Biomedical Sciences, Florida Atlantic University, Boca Raton, FL, United States

**Keywords:** angiogenesis, autoimmunity, semaphorins, MMPs, EMMPRIN, TRPs, YKL-39, VEGF

Angiogenesis is the process where new blood vessels sprout from existing ones to address increased demand for oxygen and nutrients. Angiogenesis is physiologically required in development and wound healing, and is pathologically associated with many chronic inflammatory diseases, autoimmune diseases, and cancer (1). Within the tumor or inflamed microenvironment, epithelial, or cancer cells interact with stromal cells to determine which factors are secreted and to what extent. The balance between pro- and anti-angiogenic factors determines neovascularization. When pro-angiogenic factors are in excess over anti-angiogenic ones, the “angiogenic switch” is turned on resulting in the activation, proliferation, and migration of endothelial cells, and their spatial organization as new blood vessels. These vessels that feed the tissue are often leaky and permeable (2), and enable the infiltration of immune cells to the site, promoting a state of chronic inflammation.

Interventions designed to block angiogenesis were developed and some are in clinical use. Vascular endothelial growth factor (VEGF) or its receptors are targeted using monoclonal antibodies or small molecule tyrosine kinase inhibitors (3, 4). However, too often inhibition was transient, accompanied by off-target toxicities and a “rebound effect” of enhanced disease progression upon treatment withdrawal. This highlighted the redundancies of pro-angiogenic factors and the activation of compensatory mechanisms (5), and exemplified the complexity of the system, and therefore requiring new and more efficient strategies. This Research Topic provides an updated overview of new pro-angiogenic molecules and approaches to target familiar molecules.

First, the advantages of using active peptide vaccination against angiogenic targets is reviewed by Rahat. This strategy is generally considered a simple approach, with high specificity, reduced costs, easy synthesis, safe, and well-tolerated in comparison to traditional use of monoclonal antibodies against such targets. However, this strategy did not yield significant clinical benefits, and the review discusses reasons for this failure, including the choice of target, the type of peptides, the adjuvants, and the delivery methods used. This analysis is then followed by practical recommendations for peptide vaccinations.

The extracellular matrix (ECM) consisting of basement membrane (BM) and the underlying stroma plays an important role in angiogenesis. Members in the family of matrix metalloproteinases (MMPs), MMP-9, MMP-14, and MMP-2 that are strongly associated with angiogenesis, degrade the ECM allowing migration of endothelial cells. Fields describes different classes of selective MMP inhibitors, including antibodies and their fragments, triple-helical peptides, and small molecule compounds, developed specifically against these three MMPs and the principle of their inhibitory activity. Since MMPs can also activate anti-angiogenic factors (e.g., angiostatin, endostatin) that promote vessel normalization and/or regression, Fields reminds us that the correct timing or “window of opportunity” for the use of such inhibitors should be carefully determined.

Smani et al. explored the role of transient receptor potential (TRP) channels expressed by endothelial cells in growth-factor-induced angiogenesis. TRP channels are activated by pro-angiogenic factors resulting in rise of intracellular ions such as Ca^2+^ and activation of signaling pathways that promote angiogenesis. Thus, selective pharmacological TRP channel blockers may be additional strategies for anti-angiogenic therapies.

Angiogenesis is closely associated with intracranial aneurysm recurrence after surgery using the stent-jailing and stent-jack techniques. Exploring the difference between these two techniques, Xu et al. show that stent-jack causes higher mechanical forces in cerebral vessels than stent-jailing. They demonstrate lower micro-vessel density, TGFβ and Smad 2, 3, and 4 levels in the stent-jailing group compared to the stent-jack group, and conclude that the choice in surgical technique of stent-jailing could reduce shear stress, TGFβ signaling, and angiogenesis.

The role of angiogenesis in autoimmune diseases is beginning to unfold, and new approaches to its targeting are described in the next set of papers. Iragavarapu-Charyulu et al. review the role of different classes of semaphorins, axonal guidance molecules, with respect to their angiogenic activity and autoimmunity. Classes 3, 4, and 5 mediate either angiogenic or anti-angiogenic effects by signaling through neuropilins or plexins, and class 7 mediate angiogenic effects through binding to β1-integrin and Plexin-C1. Different strategies to target semaphorins to control angiogenesis and autoimmune diseases are addressed in this paper. In another paper, Adi et al. demonstrate that administration of Semaphorin 3A in an ovalbumin-induced mouse model of allergic asthma effectively reduced lung angiogenesis, eosinophil infiltration of lung bronchioles and arteries, and inflammatory cells in broncho-alveolar lavage. Another approach to targeting angiogenesis is demonstrated by Simanovich et al. A novel prophylactic peptide epitope was used to vaccinate against the pro-angiogenic protein EMMPRIN/CD147 in a murine model of DSS-induced colitis which mimics human Ulcerative Colitis. This vaccine resulted in improved clinical symptoms, reduced EMMPRIN expression and suppression of angiogenesis.

The critical role of angiogenesis in cancer is reviewed in the next three papers. In a mini-review, Barbosa de Aguiar and Zveiter de Moraes provide a perspective on targeting VEGF with bevacizumab, a humanized monoclonal antibody against VEGF, as both an angiogenesis inhibitor and modulator of the immune response in the tumor microenvironment, and discuss the contribution of the Fc and Fab domains of the antibody to this effect. Another family called chitinase-like proteins are glycoproteins whose levels are elevated in cancer patients and associated with poor prognosis. YKL-39 (chitinase 3-like protein 2), produced by tumor-associated macrophages (TAMs), is chemotactic for monocytes and stimulates angiogenesis. Kzhyshkowska et al. report that YKL-39 expression in tumors was found to be prognostic for metastasis after neoadjuvant chemotherapy in patients with breast cancer, suggesting YKL-39 as a target for anti-angiogenesis therapy. In the last paper, Huijbers et al. used a different approach to target CD99, a protein expressed by activated endothelial cells. Use of a conjugate vaccine that induced antibodies against CD99, resulted in reduced tumor micro-vessel density and functionality, and inhibition of tumor growth in two tumor models with high and low CD99 expression.

This collection of articles shows the tremendous diversity of pro-angiogenic molecules orchestrating angiogenesis in autoimmune diseases and in cancer, contributing to disease progression. To find cures for these diseases, new targets, and new approaches are required, and this collection suggests some new and exciting therapeutic possibilities.

## Author Contributions

All authors listed have made a substantial, direct and intellectual contribution to the work, and approved it for publication.

## Conflict of Interest

The authors declare that the research was conducted in the absence of any commercial or financial relationships that could be construed as a potential conflict of interest.
